# Innovative HIV care strategies and health system resilience in conflict-affected settings

**DOI:** 10.1186/s13031-026-00757-6

**Published:** 2026-01-30

**Authors:** Swase Dominic Terkimbi, Patrick Maduabuchi Aja, Joseph Kibirige, Buyinza Nicholas, Ejike Daniel Eze, Afodun Adam Moyosore, Nancy Bonareri Mitaki, Elna Owembabazi, Samiah Shahid, Regan Mujinya

**Affiliations:** 1 Department of Biochemistry, Kampala International University, Ishaka, Uganda; 2 Department of Physiology, Equator University of Science and Technology, Masaka, Uganda; 3 Department of Physiology, Kampala International University, Ishaka, Uganda; 4 Department of Human Anatomy, Kampala International University, Ishaka, Uganda; 5 Institute of Molecular Biology and Biotechnology (IMBB), Research Centre for Health Sciences (RCHS), The University of Lahore, Lahore, Pakistan; 6 Department of Pharmacy, Faculty of Health Sciences, Victoria university, Kampala, Uganda; 7 Department of Physiology, School of Medicine, Kabale University, Kabala, Uganda; 8 Department of Anatomy and Cell Biology, Faculty of Health Sciences, Busitema University, Tororo, Uganda; 9 Associate Faculty, College of Health Sciences, Makerere University, Kampala , Uganda

**Keywords:** HIV, Conflict zones, ART access, Health system disruption, Mental health integration

## Abstract

Armed conflict disrupts HIV care by damaging health infrastructure, displacing populations, interrupting supply chains, and increasing HIV transmission risks. This study examines structural barriers to HIV service delivery in conflict-affected settings and explores adaptive strategies to sustain care. A narrative review approach was used to provide evidence on innovative treatment and prevention models, mental health integration, and policy responses. Key findings show that governance collapse, ART stockouts, workforce shortages, and disrupted data systems undermine HIV care continuity. However, differentiated service delivery, mobile clinics, long-acting injectable ART, community-based models, digital tools, and trauma-informed approaches have shown effectiveness in fragile environments. Integrating mental health into HIV programs addresses the syndemic burden of conflict-related trauma and poor treatment outcomes. Strengthening health systems’ resilience, improving donor coordination, and aligning humanitarian and HIV strategies are essential to ensuring uninterrupted care. Delivering HIV services in conflict zones requires flexible, context-specific, and rights-based approaches. A coordinated, multisectoral response is critical to close equity gaps and advance global HIV goals.

## Introduction

Armed conflicts are growing into a global threat and security concern, particularly in the control and treatment of chronic conditions like HIV/AIDS [1]. The destruction of healthcare infrastructure, displacement of healthcare personnel, disruption of supply chains, and collapse of governance impair affected populations’ access to life-saving antiretroviral therapy (ART) [2]. According to UNAIDS, over 39 million people were living with HIV globally in 2023, with approximately 6 million lacking access to antiretroviral therapy (ART) [3]. A significant number of these treatment gaps have been reported to be associated with fragile and conflict-affected settings. In War-torn regions where hospitals and clinics are damaged or repurposed for military use, supply chains for ART and diagnostics are interrupted. Conflict promotes migration, with current estimates that over 114 million people are forcibly displaced worldwide, including refugees and internally displaced persons (IDPs) [4]. These populations often reside in overcrowded camps or informal settlements where HIV services are either insufficient or absent. Treatment disruption among displaced individuals is common and has been linked to increased viral rebound, disease progression, and emergence of drug-resistant HIV strains. Compounding these challenges are increased risk factors for HIV transmission in conflict zones, including sexual violence, transactional sex, injection drug use, and a breakdown in preventive services like condom distribution and harm reduction. Stigma and discrimination further limit healthcare-seeking behavior, particularly among women, adolescents, and marginalized groups [5]. In response, there is an urgent need for the implementation of HIV service delivery models that are not only resilient to systemic disruptions but also specifically designed to function within settings of insecurity, displacement, and humanitarian crisis. In response to these complex challenges, there is an urgent need for HIV service delivery models that are resilient to systemic disruption and tailored to conditions of insecurity, displacement, and humanitarian crisis. This paper provides a synthesized overview of the structural, clinical, and psychosocial barriers shaping HIV care in conflict settings and highlights emerging models that sustain treatment in disrupted environments. Unlike previous reviews with narrower focus [6, 7], it offers a multisectoral perspective by integrating HIV service delivery, mental health considerations, and health system resilience. The proposed conceptual frameworks further clarify how conflict-driven trauma, service disruption, and HIV risk interact, outlining key priorities for future operational research in humanitarian contexts.

## Methodology

This study adopted a narrative review approach to synthesize diverse evidence on HIV care in conflict-affected and humanitarian settings. A narrative design was selected to allow flexible integration of clinical studies, public health research, program evaluations, and policy reports on the complex and context-specific nature of HIV service delivery in fragile environments. Literature was identified through structured searches of PubMed, Scopus, Web of Science, and Google Scholar using keywords including *“HIV*,*” “conflict zones*,*” “humanitarian settings*,*” “armed conflict*,*” “displacement*,*” “ART disruption*,*” “mental health*,*” “long-acting ART*,*”* and *“health system resilience.”* Publications from 2010 to 2024 were prioritized to capture contemporary HIV responses during large-scale ART expansion. Article selection was based primarily on relevance. Priority was given to empirical studies, programmatic reports, and policy analyses that addressed HIV service delivery, treatment continuity, prevention, mental health, or health system functioning in conflict-affected setting. Grey literature from international agencies and humanitarian organizations was included to capture operational data not consistently represented in peer-reviewed journals. Although the review was global in scope, greater emphasis was placed on regions with high HIV burden and prolonged conflict, particularly sub-Saharan Africa, the Middle East, Eastern Europe, and parts of Asia. Evidence was synthesized thematically across clinical, psychosocial, and health system domains to identify common challenges, adaptive strategies, and emerging innovations for HIV care in conflict settings.

## Structural barriers to HIV care in conflict zones

**Fig. 1:**
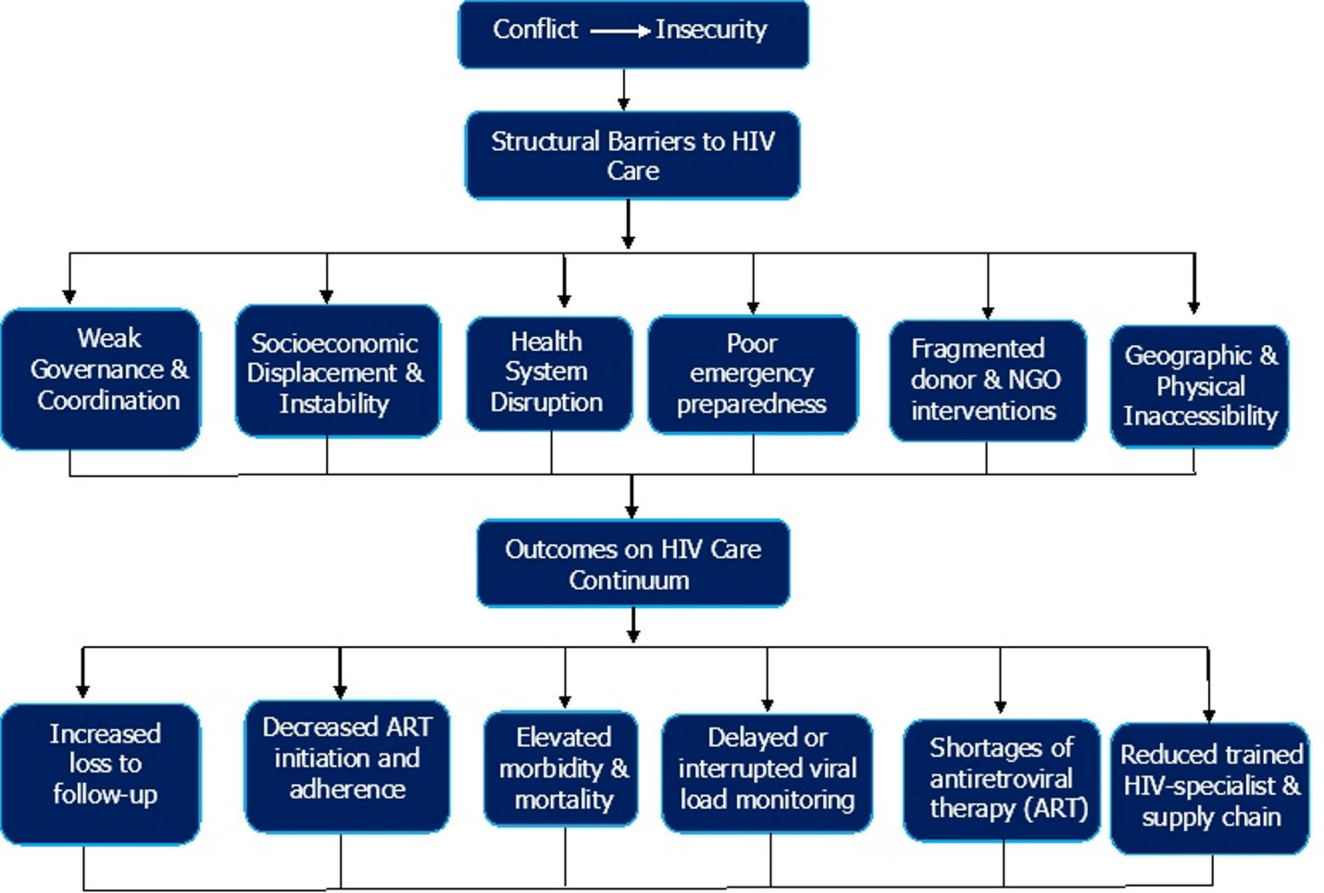
Structural determinants of HIV care disruption in conflict-affected settings

HIV service delivery in conflict zones is severely affected by systemic breakdowns that affect all aspects of the health system. These barriers are not isolated challenges but represent a collapse of the interconnected structures that affect effective healthcare services. Governance and leadership are the first casualties of war, where ministries of health lose functional authority over contested territories. This was evident in Libya, where the post-2011 conflict disrupted the majority of the HIV response infrastructure, despite increasing infections among migrants and displaced populations [8]. Without coordinated healthcare leadership or emergency health policies tailored to fragile environments, the response is left to humanitarian organizations operating without national integration or sustainability. The physical structure, like hospitals and clinics, can be destroyed, occupied by armed groups, or rendered inaccessible due to shifting frontlines. In Yemen, frequent airstrikes destroyed healthcare facilities, and insecurity prevented PLHIV from accessing routine care. Supply chains for antiretrovirals and diagnostic tools are similarly destabilized. ART stockouts and treatment interruptions are common outcomes of port closures, roadblocks, and embargoes. In Ethiopia’s Tigray region, widespread ART shortages during the 2021 conflict were reported, severely compromising treatment continuity. Cold chain requirements for certain HIV and co-infection diagnostics, such as tuberculosis or cryptococcal antigen testing, are often impossible to maintain, limiting integrated service delivery [9]. Furthermore, conflict settings are often characterized by damaged data systems and shortages of trained personnel for reliable data collection, management, and analysis. In displaced populations, clinical records are often lost or fragmented. This invisibility affects national HIV estimations, interrupts care continuity, and prevents donor coordination. This allows financial programs for HIV to be redirected or withdrawn. Governments in conflict zones may divert these funds toward security, while international donors may stop support due to instability or sanctions [10]. Additionally, Legal and governance conditions influence the continuity of HIV services during conflict. Displacement leads to loss of identification documents, limiting access to national HIV programs and free treatment. Refugees and internally displaced persons may also face legal restrictions related to residency status, border controls, or health insurance eligibility, which disrupt ongoing care [11]. In some settings, weak governance and unclear authority between humanitarian actors and national health systems create gaps in service delivery, patient tracking, and drug supply management. Criminalization of key populations and HIV-related stigma further discourage care-seeking in insecure environments. Integrating legal support into HIV programs such as assistance with documentation, protection against discrimination, and referral to legal aid can help reduce these barriers [12]. These complex and interconnected disruptions are illustrated in Fig. 1, which presents a conceptual framework of the structural barriers to HIV care in conflict zones. The figure highlights how conflict-induced insecurity triggers cascading failures across governance, health infrastructure, supply chains, and data systems. Ultimately, compromises the HIV care continuum through reduced ART access, loss to follow-up, and weakened service delivery capacity.

This conceptual framework illustrates how conflict and resultant insecurity contribute to a range of structural barriers that disrupt HIV care. These barriers include weak governance, socioeconomic displacement, health system breakdown, poor emergency preparedness, fragmented donor interventions, and physical inaccessibility. Collectively, they adversely affect outcomes along the HIV care continuum, leading to increased loss to follow-up, decreased ART initiation and adherence, delayed viral load monitoring, shortages of antiretroviral therapy, and a reduction in trained personnel.

## Syndemic intersection of HIV, mental health, Trauma, and health system disruption in conflict zones

**Fig. 2:**
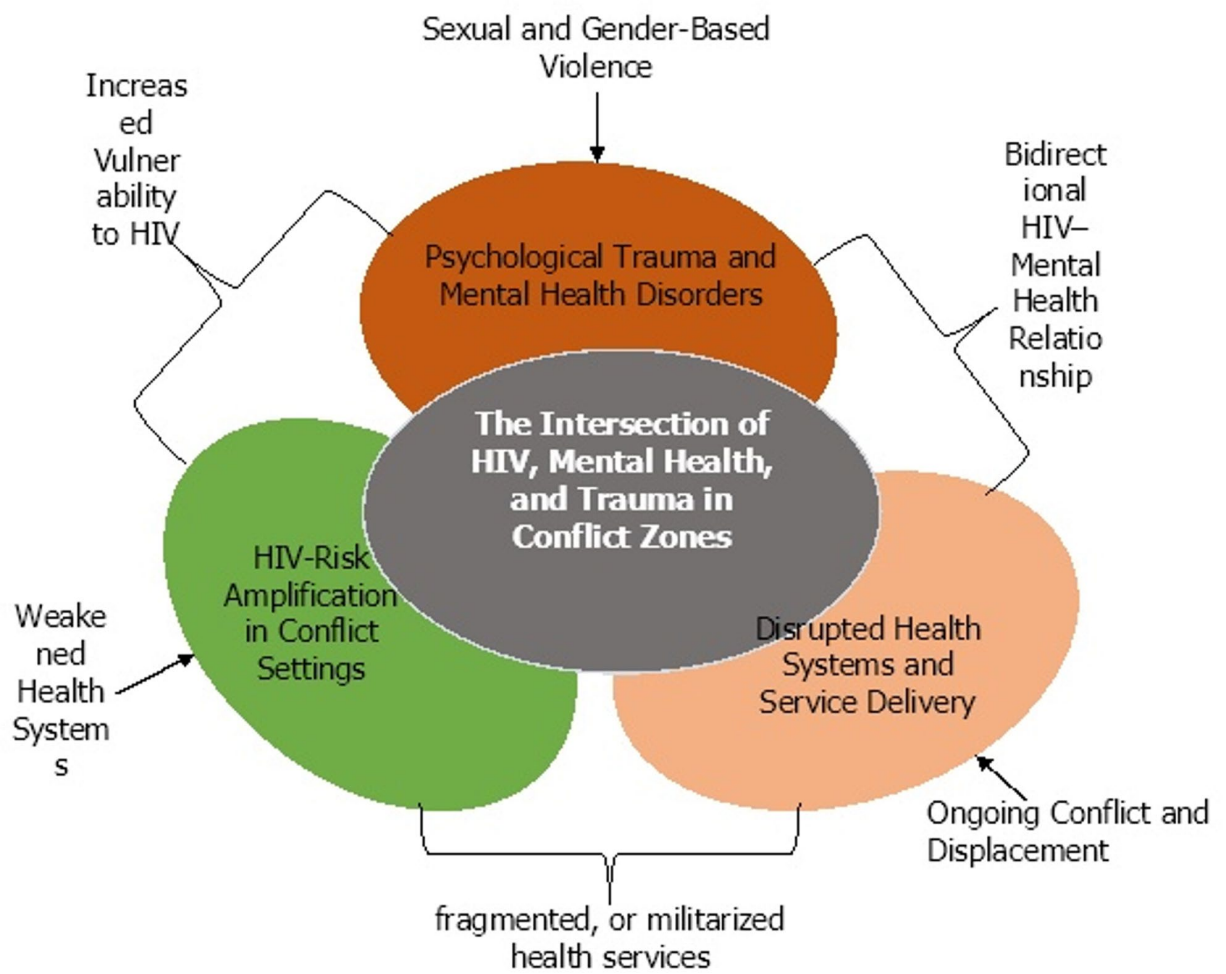
Conceptual framework illustrating the syndemic intersection of HIV, mental health, trauma, and health system disruption in conflict zones

Despite well-documented evidence linking mental illness with poor HIV treatment outcomes, mental health services remain under-integrated into HIV care in conflict zones. This systemic gap affects both individual health outcomes and public health efforts to control HIV transmission in conflict-affected settings [13]. HIV and mental health operate as syndemics, mutually reinforcing epidemics that worsen one another. Refugees and IDPs experience high rates of depression, anxiety, post-traumatic stress disorder (PTSD), and substance use disorders [14]. This can be a result of forced displacement, sexual violence, loss of family members, disrupted livelihoods, and exposure to armed conflict. These conditions directly compromise HIV outcomes by reducing treatment adherence, increasing risk-taking behaviors, delaying care-seeking, while enhancing immune suppression. Recent studies have reported the prevalence of severe depression, which correlated with poor ART adherence and loss to follow-up among HIV-positive refugees in Ethiopia, Kenya, and Uganda [15, 16]. Moreover, stigma, both HIV-related and mental health-related, further discourages displaced individuals from seeking help. In many refugee settings, psychological distress is normalized or misunderstood as spiritual weakness, while HIV status remains deeply stigmatized. Figure 2 illustrates the conceptual framework highlighting the syndemic relationship between increased HIV risk, psychological trauma, and weakened health systems in conflict zones. Sexual violence, displacement, and limited access to prevention and treatment services have all been reported to be associated with increased vulnerability to HIV infection. Concurrently, exposure to war-related stressors, including violence, loss, and insecurity, leads to a high prevalence of mental health disorders such as depression, anxiety, and post-traumatic stress disorder. These conditions are compounded by the breakdown of healthcare infrastructure, which impedes continuity of care and access to integrated services. The intersection of these three factors reflects a synergistic burden, emphasizing the urgent need for context-specific, integrated interventions that address the multifaceted health needs of populations in humanitarian crises.

The figure illustrates the overlapping and amplifying effects of three major factors in conflict-affected settings: HIV risk amplification, driven by sexual violence, displacement, and disrupted prevention; psychological trauma and mental health disorders, including depression, PTSD, and anxiety arising from conflict-related stressors; and disrupted health systems and service delivery, which disrupt access to HIV and mental health care. At the center lies the syndemic intersection, where these factors converge to promote individual vulnerability and undermine public health outcomes.

## Adaptive and innovative models for HIV care in fragile contexts

HIV Treatment in conflict zones requires more than temporary relief, it requires the reconfiguration of service models to sustain care in the absence of functioning health systems as depicted in Table 1. Community-based ART delivery is among the most common innovations addressing access challenges in conflict zones. Models such as Community ART Groups (CAGs), first established in Mozambique and later adapted to war-affected regions in South Sudan and the Central African Republic. This model functions by redistributing health responsibilities from health professionals to patients themselves [17]. These self-managed groups rotate ART collection and provide peer support, minimizing the need for frequent clinic visits and protecting individuals from travel-related risks. Evidence from Médecins Sans Frontières (MSF) programs indicates high retention rates and improved adherence under this approach, even in settings with poor facility access and frequent displacement. Additionally, mobile HIV clinics have also been employed as important lifelines in conflict zones to deliver HIV care services [18]. Mobile clinics offer decentralized access to testing, counseling, ART initiation, and sometimes point-of-care diagnostics. Their value is most apparent in rural or insecure areas of the Democratic Republic of Congo, Ukraine, and the Sahel, where static health infrastructure is non-functional or absent. Although often limited in scope, these mobile services increase visibility and access for displaced populations and those vulnerable to HIV infection due to sexual violence [19]. The application of differentiated service delivery (DSD) frameworks, particularly multi-month dispensing (MMD), offers another option for care adaptation. By supplying ART for three to six months at a time, MMD reduces the logistical burden on both health systems and patients. In northern Nigeria, pilot programs demonstrated that MMD reduced clinic congestion, improved treatment adherence, and minimized the risk of interruption during periods of insecurity. Digital tools have shown potential in conflict zones when tailored to the local digital landscape [20]. Telemedicine platforms enable virtual consultations between healthcare providers and patients, reducing the need for physical presence in insecure areas. These platforms support remote ART prescription renewals, treatment adherence monitoring, and management of opportunistic infections [21]. In addition, mobile health (mHealth) applications offer real-time health education, appointment reminders, and medication adherence tools, even in settings with limited healthcare access. For example, SMS-based adherence support programs have been used effectively among displaced PLHIV in sub-Saharan Africa, enabling ongoing communication between patients and providers [22]. Drones and other unmanned aerial vehicles (UAVs) represent a breakthrough in health logistics during emergencies. In hard-to-reach or high-risk regions, drones have been used to transport antiretroviral drugs, dried blood spot samples for viral load testing, and HIV diagnostic kits, circumventing blocked roads and conflict zones. These tools are used in conjunction with mobile clinics to extend services directly into the affected populations [23]. Mobile phone platforms ranging from SMS reminders to secure messaging apps have been used in Gaza, Syria, and eastern Myanmar to schedule appointments, facilitate adherence support, and trace defaulters [24]. While challenges such as phone ownership, internet coverage, and surveillance risks persist, digital communication enables a minimal continuity of contact between patients and healthcare providers. Task-shifting, whereby non-physician cadres are trained to provide ART services, has become a pragmatic necessity in contexts of workforce scarcity [10]. In eastern Democratic Republic of Congo and parts of Yemen, nurses, community health workers, and trained laypersons have assumed roles in ART initiation and follow-up, supported by simplified protocols and remote supervision. While clinical quality assurance remains a concern, these models have demonstrated feasibility in contexts where physicians are unavailable and traditional referral chains are disrupted [25].

**Table 1:** Adaptive and innovative models for HIV care in conflict-affected settings

Model/Strategy	Description	Adaptation in conflict settings	Advantages	Disadvantages
Community ART Groups (CAGs)	Self-managed patient groups that rotate ART collection and provide peer support	Reduces the need for frequent clinic visits; supports care continuity during displacement	Improves adherence, empowers communities, and reduces transport burden	May exclude newly diagnosed patients; requires group cohesion
Mobile HIV Clinics	Clinics on wheels offering testing, counseling, ART, and diagnostics	Delivers services to displaced or remote populations where clinics are unavailable	Increases access in insecure areas; offers integrated care	Limited by security, fuel, or road access, operational costs may be high
Differentiated Service Delivery (DSD)/Multi-Month Dispensing (MMD)	Dispensing ART for 3–6 months to reduce clinic dependence	Minimizes service disruption during insecurity or transport challenges	Reduces clinic burden, improves convenience, and enhances treatment continuity	May risk medication misuse; needs a reliable ART supply
Digital Tools for HIV Care	Mobile phones are used for SMS reminders, secure messaging, and tracking	Maintains patient-provider contact remotely during conflict	Improves adherence, enables remote support, and follow-up	Requires phone access and network coverage; data privacy concerns
Mobile Health Apps	Apps providing HIV education, reminders, and virtual assistance	Enables self-care and psychosocial support during displacement or clinic closures	Enhances autonomy, scalable support system, and user-friendly platforms	Digital illiteracy or phone access may limit use, and data usage costs
Drones for Medication Delivery	Unmanned aerial vehicles transporting ART and diagnostics	Bypasses conflict zones and roadblocks to deliver supplies safely	Rapid delivery to remote/inaccessible areas reduces supply interruptions	High initial cost; weather or airspace restrictions may apply
Task-Shifting and Decentralized Care	Training non-physicians to deliver HIV services	Sustains ART delivery when health professionals are scarce or displaced	Expands service coverage; builds local capacity	Requires training and supervision; variable quality of care
Peer-Led Outreach and Support	HIV-positive peers support adherence, counseling, and education	Utilizes trusted community members to reach mobile or marginalized groups	Reduces stigma; increases trust and retention in care	Peers may need ongoing support, risk of burnout, or role fatigue

## HIV prevention and biomedical interventions in conflict zones

The traditional antiretroviral therapy (ART) models are reliant on consistent drug availability, routine laboratory monitoring, and frequent clinical interactions. These conditions are difficult to sustain in conflict zones where accessibility and availability present a major challenge. Conflict zones require ART regimens with minimal monitoring, less toxicity, and can be administered with limited clinical supervision. Recent progress in ART optimization has led to the development of fixed-dose combinations (FDCs), which reduce drug burden and improve adherence as presented in Table 2. Dolutegravir-based regimens, particularly the tenofovir/lamivudine/dolutegravir (TLD) combination, offer high genetic barriers to resistance, favorable safety profiles, and once-daily dosing [26]. In Mozambique and the Democratic Republic of Congo, TLD-based regimens have been successfully used in mobile clinics and community ART groups. This ART regimen can offer high rates of viral suppression with limited laboratory and healthcare access. Simplified ART regimens can also reduce dependency on cold chain storage and advanced diagnostics, which are often unavailable in conflict zones [27]. One of the most transformative innovations in HIV care for conflict-affected settings is the use of long-acting injectable ART (LA-ART). Drugs such as cabotegravir and rilpivirine, administered monthly or bimonthly, offer an important advantage. These ART regimes reduce the need for frequent clinical visits, drug storage, and adherence monitoring. For displaced individuals who may lack secure shelter, consistent access to healthcare, or privacy to store drugs, injectable ART can mitigate many logistical and social barriers [28]. Building on the benefits of long-acting injectable ART, lenacapavir offers a further advantage for HIV care in conflict-affected settings. Lenacapavir is a first-in-class capsid inhibitor administered subcutaneously every six months, making it the longest-acting HIV treatment currently available. This extended dosing interval greatly reduces the need for frequent clinic visits, medication storage, and adherence monitoring. Such features are particularly valuable in settings affected by insecurity, displacement, and health system disruption. Early studies suggest that lenacapavir can maintain viral suppression among individuals with unstable treatment access or prior interruptions, supporting continuity of care where routine HIV services are difficult to sustain [29]. Prevention of new HIV infections remains a top priority in conflict zones, where sexual violence, transactional sex, and disrupted health services amplify transmission. Post-exposure prophylaxis (PEP) and pre-exposure prophylaxis (PrEP) are important prevention tools that can be adapted for humanitarian settings with the right infrastructure and policy support [30]. Oral PrEP, such as tenofovir/emtricitabine, has been successfully distributed through mobile clinics and refugee health centers in Kenya, Uganda, and Lebanon [31]. Simplified delivery of pre-exposure prophylaxis (PrEP) through multi-month dispensing, peer-led distribution, and community-based models can improve prevention coverage in unstable settings. Post-exposure prophylaxis (PEP) should be routinely integrated into post-rape care within humanitarian health services, alongside emergency contraception and mental health support. In conflict settings with ongoing risk of sexual violence, oral PrEP has also been incorporated into sexual and gender-based violence (SGBV) response kits, representing an important innovation in HIV prevention [32]. Microbicides such as the dapivirine vaginal ring offer a discreet, female-controlled option for HIV prevention in high-risk settings. Although still under limited rollout, these products have shown promise in empowering women in contexts where negotiating condom use is difficult or impossible [33]. Lenacapavir is a newer antiretroviral drug with particular relevance for conflict-affected settings. It is given as an injection every six months, which reduces the need for daily pill-taking and frequent clinic visits. This long dosing interval is especially useful where displacement, insecurity, and weak health systems disrupt regular care [34]. Lenacapavir may complement other long-acting antiretroviral therapies by offering the longest dosing interval currently available. However, expanding access in conflict settings remains difficult due to declining donor funding and unstable supply chains, which also affect the availability of PrEP and PEP [35].

**Table 2:** Biomedical HIV prevention innovations for conflict-affected populations

Intervention	Description	Adaptation in conflict settings	Advantages	Disadvantages
Fixed-Dose Combinations (FDCs)	Combines multiple ART drugs into a single pill (e.g., TLD, tenofovir/lamivudine/dolutegravir)	Suitable for mobile clinics and community-based distribution; minimal laboratory monitoring required	Reduces pill burden, improves adherence, high genetic barrier to resistance	Limited options for patients with drug resistance or specific contraindications
Long-Acting Injectable ART (LA-ART)	Monthly or bimonthly injectable ART (e.g., cabotegravir/rilpivirine)	Well suited for displaced or highly mobile populations with difficulty maintaining daily adherence	Eliminates daily pill-taking, improves privacy, reduces stigma, and supports adherence	Requires cold chain, trained personnel, and regular injection visits, missed doses may risk resistance
Lenacapavir (Ultra-Long-Acting ART)	First-in-class capsid inhibitor administered subcutaneously every six months	Highly suitable for conflict settings with severe access constraints and frequent service disruption	Very infrequent dosing, minimal adherence burden, reduced clinic visits, no daily storage needs	Limited availability, higher cost, and currently restricted to specific treatment indications
Oral Pre-Exposure Prophylaxis (PrEP)	Daily oral medication (e.g., tenofovir/emtricitabine) for HIV-negative individuals at high risk	Delivered via mobile clinics, community health workers, or peer-led outreach in conflict zones.	High efficacy, scalable, allows multi-month dispensing.	Requires daily adherence; may face stigma or poor understanding.
Post-Exposure Prophylaxis (PEP)	28-day oral regimen initiated after potential HIV exposure	Integrated into humanitarian emergency and sexual violence response services	Critical for preventing HIV after high-risk exposure	Must begin within 72 h and requires full course completion
Dapivirine Vaginal Ring (Microbicide)	Vaginal ring releasing antiretroviral drug over 28 days for HIV prevention	Useful where women have limited power to negotiate condom use; discreet and user-controlled	Empowers women, no daily action required, low systemic exposure	Requires monthly replacement; limited availability and awareness

## Policy implications and health resilience system

**Fig. 3:**
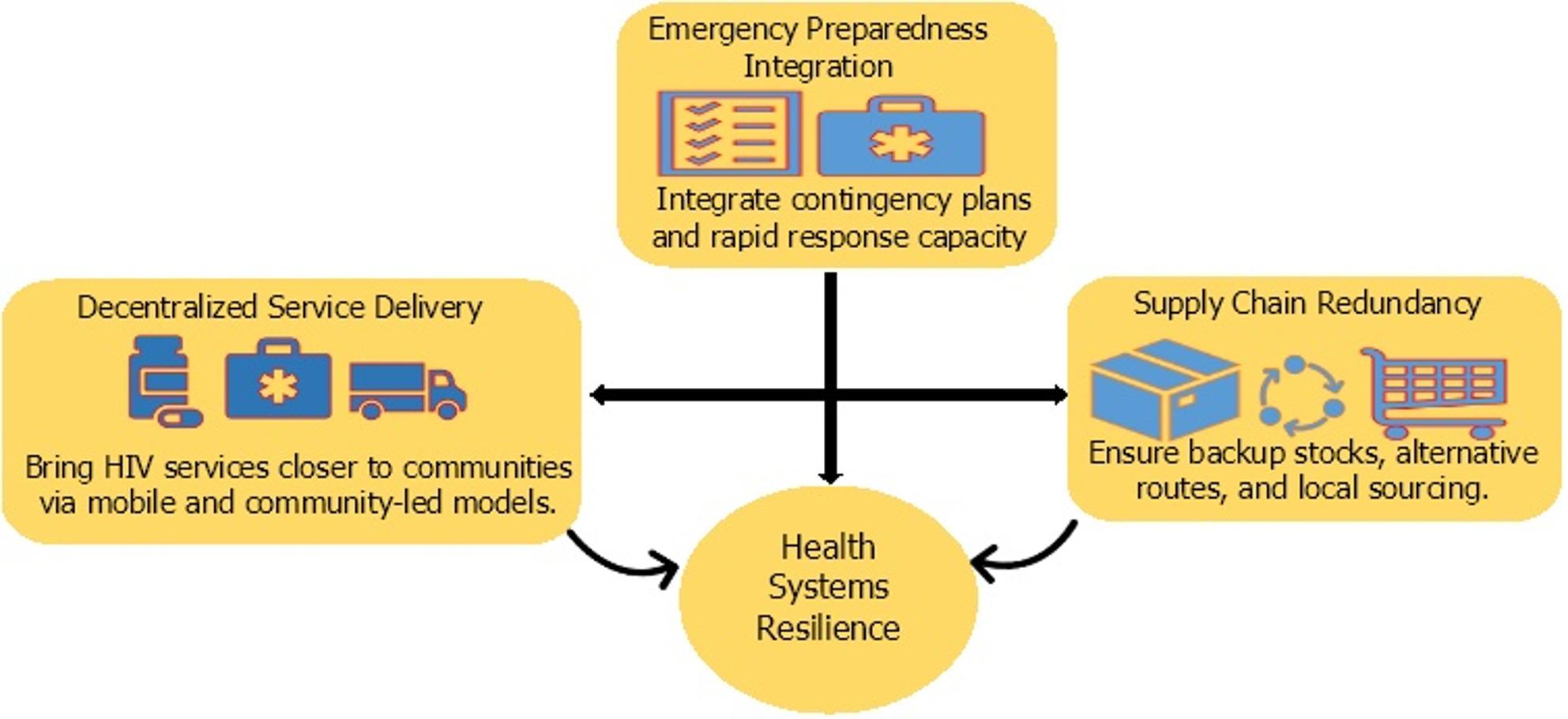
Health systems resilience and multisectoral coordination in HIV response

Resilience requires forward-looking, building adaptive systems capable of functioning amid uncertainty during conflicts. This includes integrating emergency preparedness into HIV program planning, decentralizing service delivery, and embedding redundancy into supply chains as depicted in Fig. 3.

This figure outlines three core strategies essential for strengthening health systems resilience to maintain HIV service continuity in conflict-affected regions: [1] Emergency Preparedness Integration, involving the incorporation of contingency planning and rapid response mechanisms; [2] Supply Chain Redundancy, which emphasizes securing backup inventories, diversifying supply routes, and encouraging local sourcing to prevent stockouts; and [3] Decentralized Service Delivery, aimed at expanding access through mobile and community-led HIV care models.

Ministries of Health must collaborate with humanitarian organizations, local NGOs, and emergency response agencies. In South Sudan, for example, partnerships with Médecins Sans Frontières (MSF) helped maintain HIV treatment during displacement through mobile ART delivery and outreach programs. National responses should also align with global humanitarian frameworks such as the Inter-Agency Standing Committee (IASC) Health Cluster [36]. This coordination reduces duplication, addresses service gaps, and promotes accountability. Developing resilient health systems is essential for sustaining care during conflict. This includes decentralizing ART distribution, training community-level providers, and strengthening health information systems. Donor harmonization is equally important where fragmented funding and short-term grants often disrupt HIV programs. In Yemen, inconsistent donor support led to ART stockouts and treatment interruptions. Donors should coordinate with national authorities to ensure consistent, long-term funding that integrates HIV services into broader emergency health and recovery frameworks [37]. Successful examples include aligned efforts by PEPFAR, UNHCR, and the Global Fund in conflict-affected regions. Adopting these policy approaches can safeguard HIV treatment access in unstable settings. They also contribute to stronger, more flexible health systems capable of enduring future crises, supporting global goals such as epidemic control and universal health coverage [38]. Effective HIV programming in conflict zones requires simultaneous engagement from sectors including security, humanitarian aid, social protection, gender-based violence (GBV) prevention, education, and civil society. Lessons from the Democratic Republic of Congo illustrate how coordinated responses between HIV programs, sexual violence response units, and maternal health services can be. This can be used to address overlapping vulnerabilities among conflict-affected women and girls [39]. Its embeddedness in local networks, cultural familiarity, and relative mobility allows it to sustain outreach, provide psychosocial support. Conflict-sensitive health information infrastructure capable of functioning offline, with simplified data collection and encryption, can enable case tracking, adherence monitoring, and population-level surveillance even in insecure regions. The use of open-source platforms such as DHIS2, adapted for offline use and employed by health workers through mobile tablets, has demonstrated feasibility in conflict zones [40]. Donor alignment and governance collaborations are also essential to resilience in conflict zones. Coordination with national authorities, where feasible, and transitional health authorities in the absence of governance remains important [41]. Resilient HIV response must incorporate peacebuilding and protection objectives into its strategic design. In South Kivu (DRC), for example, integrating HIV services into peace education and reconciliation programs improved community trust and service uptake [42]. Such integrated and coordinated responses are effective in addressing overlapping vulnerabilities related to violence, displacement, and HIV risk.

A sustainable HIV response in conflict zones requires human rights protection and coordinated financing. The Geneva Conventions and their Additional Protocols obligate warring parties to ensure the protection of civilian populations and the wounded and sick, including access to necessary medical care without discrimination [43]. International human rights instruments, including the International Covenant on Economic, Social and Cultural Rights (ICESCR), further recognize the right to the highest attainable standard of health, which applies universally, including during emergencies. The UN Security Council has passed several resolutions (e.g., Resolution 1308 on HIV/AIDS and international peacekeeping, and Resolution 1983 on HIV in conflict settings) that acknowledge the intersection of HIV, conflict, and peacebuilding [44].

## Integration of mental health and Trauma-Informed care (TIC) into HIV care

The convergence of HIV, forced displacement, and exposure to war-related trauma creates a syndemic environment where mental illness becomes both cause and consequence of poor HIV outcomes. In Lebanon, mobile psychosocial teams integrated into HIV clinics have provided mental health assessments, trauma counseling, and continuity of care for Syrian refugees living with HIV [45]. Similarly, Médecins Sans Frontières (MSF) has integrated psychological first aid and lay counseling into its HIV programs in the Central African Republic, where war trauma and HIV co-morbidity are widespread [46]. Tools such as the WHO’s Mental Health Gap Action Programme (mhGAP) and the IASC guidelines provide practical frameworks for integrating mental health support into existing HIV services. These include basic screening for depression and PTSD, brief interventions such as problem-solving therapy, and referral systems for more complex cases. By embedding these services within ART distribution points, mobile clinics, and community outreach, HIV programs can reach individuals who would otherwise remain outside the mental health system [47]. Standard HIV care protocols often fail to account for the profound effects of trauma on patient behavior, trust in healthcare, and clinical engagement. Trauma-informed care (TIC) offers a paradigm shift by acknowledging the pervasive impact of violence and displacement on patients’ lives and modifying care delivery accordingly. TIC emphasizes safety, trustworthiness, empowerment, and cultural sensitivity principles that are essential for PLHIV (people living with HIV) who have experienced rape, torture, forced conscription, or other war-related abuses [48]. Operationalizing trauma-informed care in HIV settings involves training providers to recognize signs of trauma, avoid re-traumatization (e.g., through invasive questioning or rigid procedures), and create psychologically safe environments. Clinics can integrate gender-based violence (GBV) screening, survivor-centered referral pathways, peer support groups, and mental health services under one roof. In northern Uganda, community-based trauma counseling linked with HIV treatment has improved both mental health outcomes and ART adherence among war-affected youth [49]. In addition to trauma-informed care, complementary strategies are needed to ensure comprehensive mental health support in conflict-affected HIV settings. Task-shifting models allow trained nurses, community health workers, and peer counselors to deliver basic mental health screening and psychosocial support in the absence of specialists [50, 51]. Community-based and peer-led support groups further address stigma, social isolation, and treatment disengagement among displaced populations. Mental health services can also be embedded within differentiated HIV service delivery platforms, such as mobile clinics and community ART distribution points, improving continuity of care during periods of insecurity. Where feasible, low-intensity digital interventions, including phone-based counseling and SMS follow-up, offer additional mechanisms to sustain mental health support when facility access is disrupted [52].

## Conclusion

HIV care in conflict-affected settings continues to face major challenges due to insecurity, displacement, and weakened health systems. Evidence from this review shows that maintaining continuity of care is most achievable when HIV services are flexible, decentralized, and adapted to unstable environments. Community-based delivery models, mobile clinics, and long-acting treatment options offer practical solutions for sustaining care when routine health services are disrupted. Mental health and trauma significantly influence HIV treatment uptake and clinical outcomes and should be integrated into routine HIV care. Recognizing the effects of conflict-related trauma can improve trust in health services, support adherence, and enhance long-term retention in care. Future research should focus on operational and implementation studies that assess the scalability, long-term effectiveness, and sustainability of innovative HIV care models in conflict settings. Priority areas include long-acting therapies, community-led delivery platforms, digital tools adapted for low-connectivity environments, and integrated mental health services. Strengthening local capacity through training of frontline health workers and reinforcing supply-chain systems will be essential to support durable implementation. Advancing this evidence base will help refine context-appropriate strategies and guide more equitable and resilient HIV responses in humanitarian settings.

## Data Availability

No datasets were generated or analysed during the current study.

## Abbreviations

AIDS: Acquired Immunodeficiency Syndrome
ART: Antiretroviral Therapy
CAGs: Community ART Groups
DRC: Democratic Republic of the Congo
DSD: Differentiated Service Delivery
FDCs: Fixed-Dose Combinations
HIV: Human Immunodeficiency Virus
LA-ART: Long-Acting Antiretroviral Therapy
MSF: Médecins Sans Frontières
PEP: Post-Exposure Prophylaxis
PLHIV: People Living with HIV
PTSD: Post-Traumatic Stress Disorder
TIC: Trauma-Informed Care
TLD: Tenofovir, Lamivudine, and Dolutegravir
UAVs: Unmanned Aerial Vehicles
UNAIDS: Joint United Nations Programme on HIV/AIDS
UNHCR: United Nations High Commissioner for Refugees
WHO: World Health Organization
